# Medicinal flora and ethnoecological knowledge in the Naran Valley, Western Himalaya, Pakistan

**DOI:** 10.1186/1746-4269-9-4

**Published:** 2013-01-10

**Authors:** Shujaul M Khan, Sue Page, Habib Ahmad, Hamayun Shaheen, Zahid Ullah, Mushtaq Ahmad, David M Harper

**Affiliations:** 1 Department of Botany, Hazara University Mansehra, Pakistan; 2 Department of Geography, University of Leicester, UK; 3 Department of Genetics, Hazara University Mansehra, Pakistan; 4 Department of Plant Sciences, Quaid-e-Azam University, Islamabad, Pakistan; 5 Department of Biology, University of Leicester, UK

**Keywords:** Biodiversity conservation, Ecosystem services, Medicinal plants, Vegetation

## Abstract

**Background:**

Mountain ecosystems all over the world support a high biological diversity and provide home and services to some 12% of the global human population, who use their traditional ecological knowledge to utilise local natural resources. The Himalayas are the world's youngest, highest and largest mountain range and support a high plant biodiversity. In this remote mountainous region of the Himalaya, people depend upon local plant resources to supply a range of goods and services, including grazing for livestock and medicinal supplies for themselves. Due to their remote location, harsh climate, rough terrain and topography, many areas within this region still remain poorly known for its floristic diversity, plant species distribution and vegetation ecosystem service.

**Methods:**

The Naran valley in the north-western Pakistan is among such valleys and occupies a distinctive geographical location on the edge of the Western Himalaya range, close to the Hindu Kush range to the west and the Karakorum Mountains to the north. It is also located on climatic and geological divides, which further add to its botanical interest. In the present project 120 informants were interviewed at 12 main localities along the 60 km long valley. This paper focuses on assessment of medicinal plant species valued by local communities using their traditional knowledge.

**Results:**

Results revealed that 101 species belonging to 52 families (51.5% of the total plants) were used for 97 prominent therapeutic purposes. The largest number of ailments cured with medicinal plants were associated with the digestive system (32.76% responses) followed by those associated with the respiratory and urinary systems (13.72% and 9.13% respectively). The ailments associated with the blood circulatory and reproductive systems and the skin were 7.37%, 7.04% and 7.03%, respectively. The results also indicate that whole plants were used in 54% of recipes followed by rhizomes (21%), fruits (9.5%) and roots (5.5%).

**Conclusion:**

Our findings demonstrate the range of ecosystem services that are provided by the vegetation and assess how utilisation of plants will impact on future resource sustainability. The study not only contributes to an improved understanding of traditional ethno-ecological knowledge amongst the peoples of the Western Himalaya but also identifies priorities at species and habitat level for local and regional plant conservation strategies.

## Introduction

The benefits obtained by humans from nature are termed as Ecosystem Services [1,2]. Natural ecosystems provide human societies with vital supporting services, such as air and water purification, climate regulation, waste decomposition, soil fertility & regeneration and continuation of biodiversity. The Millennium Ecosystem Assessment (2003) and few other studies of ecosystem services have classified these services into four broad categories – provisioning, regulating, supporting and cultural [3-8]. These services are produced by complex interactions between the biotic and abiotic components of ecosystems. All kinds of ecosystem services, whether provisioning, regulating, supporting or cultural, are closely allied to plant biodiversity [9,10]. All sort of these services ultimately contribute to agricultural, socio economic and industrial activities [11-13]. Plant biodiversity on slope surfaces of the mountains regulates supply of good quality water and prevents soil erosion and floods. It also enhances soil formation, fertility, nutrient and other biogeochemical cycling. Provisioning services provided by plant biodiversity are in the form of food, grazing land and fodder for their livestock, fuel wood, timber wood, and medicinal products. Culturally, people utilize plants in number of ways like aesthetics, religion, education, naming etc.

People extensively utilize the predominant herbaceous flora of mountainous ecosystems by keeping cattle and multipurpose collection, both of which cause over-exploitation of the vegetation and risks to the continuation of plant biodiversity. In order to develop appropriate systems for the sustainable use of plant resources, it is crucial to understand how traditional uses of plants influence biodiversity in these ecosystems. A plant that possesses therapeutic properties or exerts beneficial pharmacological effects on the human or animal body is generally designated as “medicinal plant”. It has also been recognized that these plants naturally synthesize and accumulate some secondary metabolites, like alkaloids, glycosides, tannins, volatile oils, minerals and vitamins, possess medicinal properties [14,15]. A number of medicinal plants possess some special characteristics that make them special in those mountainous regions of the Himalayas and adjacent ranges [16,17]. Medicinal plants constitute an important natural wealth of that region and ultimately at national level. They play a significant role in providing primary health care services to rural people [18]. They serve as healing agents as well as important raw materials for the manufacturing of traditional and modern medicine [19]. Similarly a substantial amount of foreign exchange can be earned through exporting medicinal plants to other countries. In this way indigenous medicinal plants play significant role of an economy of a country. This paper therefore, sought to, not only studies the natural vegetation of the Naran Valley, but also to the indigenous people of the valley in an assessment and identification of the plant species of therapeutic uses.

### Study area

**Figure 1:**
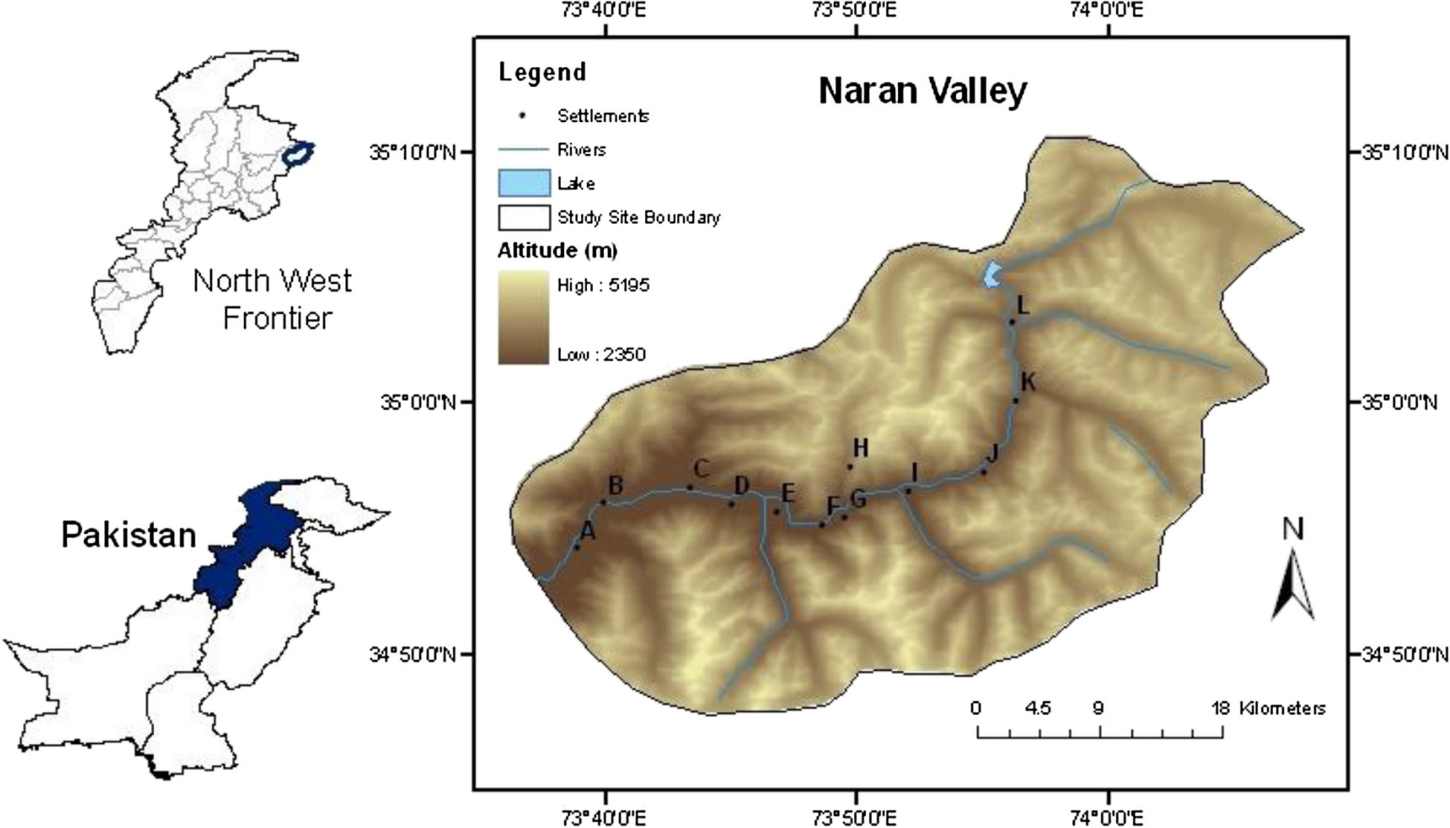
**Physiographic map (produced through Arch GIS) of the Naran Valley; elevation zones, location of its main settlements (A-L), the River Kunhar, originating lake (the Lake Lulusar) and the tributary streams.** (Elevation data obtained from the ASTER GDEM, a product of METI and NASA).

The Naran Valley, Khyber Pakhtunkhwa, Pakistanis is about 60 km long valley and can be located at 34° 54.26’N to 35° 08.76’ N latitude and 73° 38.90’ E to 74° 01.30’ E longitude; elevation between 2450 to 4100 m above mean sea level. The entire area is formed by high spurs of mountains on either side of the River Kunhar which flows in a northeast to southwest direction down the valley to the town of Naran. Geographically, the valley is located on the extreme western boundary of the Himalayan range, after which the Hindu Kush range of mountains starts to the west of the River Indus. Geologically, the valley is situated on the margin of the Indian Plate where it is still colliding against the Eurasian plate (Figure 1). Floristically, the valley has been recognised as an important part of the Western Himalayan province [20], while climatically, it has a dry temperate climate with distinct seasonal variations.

### Methodology for ethnobotanical data collection and analyses

An ethno-ecological study was carried out to explore how the local people interact with natural plant biodiversity. Interviews using questionnaires were organized during summer (May-September) 2010. Data was collected in two phases i.e., field survey and questionnaire survey.

a) Observations of local people during first fieldwork (summer, 2009) about the utilization of plant biodiversity for various purposes were used for the questionnaire preparation. A mixture of qualitative and quantitative methods of data collection was adopted in preparing a questionnaire for collecting indigenous knowledge about plant species. Local names of plants were listed along with the botanical names of the recorded 198 plant species [21,22]. Plant species photographed during the first field campaign were shown to the interviewees where and when it was felt necessary.

b) Each of the main 12 localities (A-L) in the project area (at an interval of about 5 km each), where vegetation transects had been taken, were revisited (Figure 1). Meetings were arranged with village heads or councillors and permission as well as guidance was obtained. Ethnic groups including the Gujars, Syeds, Swati and Kashmiri inhabit the valley. The most important among these are the Gujars (descendents of the Indian Arians) who are famous for their unique culture, way of life, rituals and bravery. The Gujars are concentrated in the upper parts of most valleys in Pakistan where they cultivate rain-fed slopes, and are generally more aware of traditional knowledge, of plant use and local ecology. A local community member of these tribes was taken as a guide who knew the norms and traditions of that indigenous society [23,24]. Ten houses at each of the 12 main localities of the Valley (a total of 120) were selected randomly for the interviews, using a random number table. Each village was visited from one side; a coin was tossed in front of each 5th house and if it fall head side up, then an interview was requested from that family [24,25]. If willing, one member in the household was interviewed about their uses of plants, preferences, therapeutic application and plant part that were used. Informants were asked about their general uses of plant species, e.g. as food, fodder, grazing, timber, fuel, aesthetic, medicinal and others. Respondent were then asked about their species preference if they utilised a species for several purposes – food, fodder, grazing, fuel, timber or medicinal purposes. As there was much preference for medicinal uses of plants and hence informants were further asked for details about the plant part(s) that were used, the diseases it cured and the recipe of use.

Questionnaire data was initially analyzed for basic categorization of the respondents’ gender, age groups and literacy ratio etc. This data was additionally analyzed for use preferences, plants parts used, recipes and treatment categorization with slight changes to the methodology adopted by [26-28].

## Results

### Preliminary information about the respondents

The questionnaire respondents represented a diverse array of people including farmers, women, literate, illiterate, young and elders. Among the 120 informants, 87 were male and 33 were female. The largest proportion of the respondents was of elderly, above 40 years old (81.6%) (Table 1). More than half of the respondents were illiterate (51.7%), whilst, most of those with an education had merely primary which reflect the unavailability of educational institution in the area (30%) (Table 1). These very basic results also reflect the reality that indigenous knowledge is well established but seems to be decreasing in the younger generation.

**Table 1:** Age group and literacy level frequencies of the interviewed people in the region

**Age group**	**No. of Individuals**	**Percentage**	**Literacy Level**	**No. of Individuals**	**Percentage**
14-30	6	5.0	Illiterate	62	51.7
31-40	16	13.3	Primary	36	30.0
41-50	41	34.2	Middle	11	9.2
51-60	43	35.8	Secondary	10	8.3
61-70 +	14	11.7	University	1	0.8

### Preference analysis

**Figure 2:**
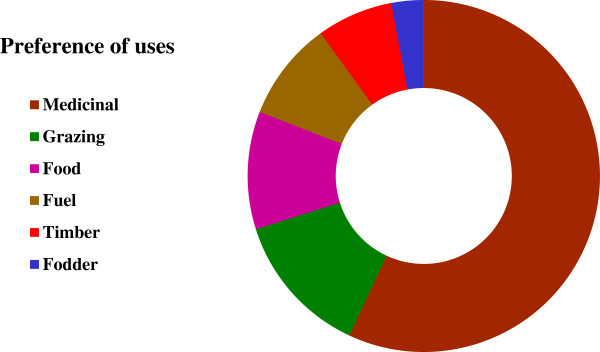
Preferences mentioned by the informants for the species having more than one local use.

Many of the recorded species (83%) provide a number of provisioning services and hence the respondents were asked what preference they gave for a specific service category. The results of preference analysis showed the highest priority of local people for medicinal use of plant species (56.9% responses) followed by grazing and food (13.1% and 10.8% respectively) (Figure 2). The high priority given to medicinal use illustrates the high level of traditional knowledge about plants in the community and the lack of basic health facilities. It can also be attributed to the high market value of medicinal species.

As people of the region preferred the plants for therapeutic purposes and hence detailed analyses were carried out on medicinal services.

### Ethnomedicinal plant resources

People in the valley use 101 species belonging to 51 families (51% of the total plants) for medicinal purposes (55.4% of the used species). Lamiaceae, with 9 species, was the most represented medicinal family followed by Polygonaceae and Rosaceae with 8 species each.

### Important medicinal plant species

Each medicinal species found in the region is noteworthy but a few of them got much importance in the local health care system e.g., *Dioscorea deltoidea* is locally used in urinary tract problems, as tonic and anthelmintic. Local hakeems (experts in traditional medicine) use *Podophyllum hexandrum*) in digestive troubles and treating cancer. Powdered bark of *Berberis pseudoumbellata* is locally utilized for the treatment of fever, backache, jaundice and urinary tract infection whilst its fruit is valued as a tonic. Orchid species i.e., *Cypripedium cordigerum* and *Dactylorhiza hatagirea* are considered as aphrodisiacs and as nerve tonics. Other noteworthy medicinal species are *Cedrus deodara* and *Aesculus indica*. Oils extracted from *Cedrus deodara* are used in skin diseases while powder of the dried fruit nuts of *Aesculus indica* are used in colic and also as worm expeller. Among other species, *Aconitum heterophyllum, Aconitum violaceum, Ephedra gerardiana, Eremurus himalaicus, Hypericum perforatum, Indigofera heterantha, Geranium wallichianum, Iris hookeriana, Nepeta laevigata, Origanum vulgare, Paeonia emodi, Rheum austral, Thymus linearis* and *Ulmus wallichiana* are also of great importance in the traditional health care. For detailed use of each species see Table 2.

**Table 2:** Plant species with their local names, part used and traditional medicinal uses

**S. N**	**Botanical Name**	**Family Name**	**Altitude (m)**	**Locality**	**Local Name**	**Part used**	**Uses**
1	*Achillea millefolium* L.	Asteraceae	2840	Bans	Birangesif/Jarri	Whole plant	Less concentrated decoction mixed with milk is taken in stomach disorders and diarrhoea.
2	*Aconitum heterophyllum* Wall.	Ranunculaceae	3250	Besal	Patris/Sarba vala	Rhizome	Pills of rhizome powder coated in local butter are used as aphrodisiac and general body tonic.
3	*Aconitum violaceum* Jacq. ex Stapf	Ranunculaceae	3310	Serrian	Atees/Zahar	Paired roots	Powders are used in sciatica and as pain killer.
4	*Actaea spicata* L.	Rosaceae	3130	Lalazar	Beenakae	Root, berries	Berries are used as sedative; Extract is applied externally for the treatment of joint pains.
5	*Adiantum venustum* D. Don	Adiantaceae	3020	Upper Batakundi	Sumbal	Whole plant	Infusion is taken orally for lungs disorders.
6	*Aesculus indica* (Wall. ex Camb) Hook.	Hippocastanceae	2460	Damdama	Bankhore/Javaz	Fruit	Powder of the dried fruit is used in indigestion.
7	*Allium humile* Kunth.	Alliaceae	3570	Lalazar Peak	Jangali piaz	Whole plant	Fresh plant is taken as salad for gastrointestinal disorders and UTI.
8	*Angelica glauca* Edgew.	Apiaceae	3410	Lalazar	Chora chora	Dried roots	Powdered roots are taken with milk for gastrointestinal disorders.
9	*Artemisia absinthium* L	Asteraceae	2800	Lalazar	Chahu/Tarkha	Flowering tops	Crushed powders are used to enhance digestion as well as worm problems.
10	*Artemisia vulgaris* L.	Asteraceae	2630	Batakundi R. Station	Chahu/Javkey	young shoots	Extract of its young shoots is used to regulate monthly cycle.
11	*Asparagus racemosus* Willd.	Asparagaceae	2770	Barrawae	Nanoor/Shalgvatey	Root & stem	Paste of powder is applied for wounds healing (Antiseptic); powders are taken orally to stimulates sexual desire and treat dysentery.
12	*Berberis pseudoumbellata* Parker	Berberidaceae	2900	Batakundi	Sumbal/Kvarey	Root, bark & fruit	Powder of roots bark is used in fever, backache, jaundice, and UTI. Fruit is considered as tonic.
13	*Bergenia ciliata* (Haw.) Sternb.	Saxifragaceae	2940	Upper Batakundi	But pewa/Zakhme hayat	Latex & Rhizome	Latex is applied externally for gum diseases and decoction of rhizome is used in kidney stones.
14	*Bergenia strachyei* (Hook. f. & Thoms) Engl	Saxifragaceae	3190	Such Peak	But pewa/Zakhme hayat	Rhizome, Latex	Latex is applied externally for gum diseases; Decoction of rhizome is used in kidney stones and for contraction of tissues.
15	*Betula utilis* D. Don	Betulaceae	3250	Such	Braj	Leaves, bark	Tea made up of young leaves is used as diuretic and joints pain; rarely used for gall bladder stone.
16	*Bistorta affinis* (D. Don) Green	Polygonaceae	3350	Lower Batakundi	Anjabar	Rhizome	Powders prepared from rhizome taken with milk in fever, body pains & muscles contraction.
17	*Bistorta amplexicaulis* (D. Don)	Polygonaceae	2680	Batakundi R. Station	Masloon	Rhizome	Powder mixed with little salt is used for sore throat, swelling of mouth and tongue.
18	*Caltha alba* Jack. ex Comb	Ranunculaceae	2960	Khora	Baringu	Roots & airial parts	Roots infusion is used as mouth wash; young shoots and leaves are cooked as vegetable for and considered as digestive.
19	*Capsella bursa-pastoris* (L.) Medic.	Brassicaceae	2540	Lower Batakundi	Chambraka	Aerial parts, seeds	Aerial parts are cooked and used in diarrhoea; Seeds powder is taken with water to cure high blood pressure.
20	*Cedrus deodara* (Roxb. ex Lamb.) G. Don	Pinaceae	2700	Naran	Diar/Ranzrra	oil	Oil are extracted from wood through burning and used to cure skin disorders.
21	*Chenopodium album* L.	Chenopodiaceae	2460	Naran	Sarmay	Leaves & shoots	Leaves and shoots are cooked and taken to expel worms also to promote evacuation of bowels and urine.
22	*Clematis montana* Buch.-Ham. ex DC	Ranunculaceae	2820	Lalazar	Zelae	Flowers & Fruits	Flowers and fruits powder is taken for treating the diarrhoea & dysentery.
23	*Colchicum luteum* Baker	Colchicaceae	3230	Bans	Qaimat-guley/Suranjane talkh	Dried corms	Very small amount of powder is given by Hakims (specialist people) in local oils as aphrodisiac and in joint pains, spleen & liver diseases.
24	*Convolulus arvensis* L.	Convolulaceae	2940	Upper Batakundi	Sahar gulay	Roots	Powder is considered as purgative & used in evacuation of bowels.
25	*Corydalis govaniana* Wall.	Fumariaceae	3310	Damdama	Desi mamera	Whole plant	Juice o the plant is used as diuretic powders of flowers are used in treating eye diseases.
26	*Cotoneaster microphyllus* Wall. ex Lindl	Rosaceae	2720	Dadar Nalah	Mamanna/Kharava	Leaves and shoots	Tea prepared from leaves is used to stop bleeding and peep.
27	*Crataegus oxycantha* L.	Rosaceae	2410	Naran	Tampasa	Fruits and flowers	Fruit and flowers are considered as heart tonic.
28	*Cypripedium cordigerum* D. Don	Orchidaceae	3050	Batakundi	Shakalkal	Rhizome	Powders are used by experts relieve spasm and as nerve stimulant
29	*Dactylorhiza hatagirea* (D. Don) Soo	Orchidaceae	2760	Saifalmaluk Nalah	Salap	Tubers	Tubers powders are used by hakims as sex stimulant & nerve tonic.
30	*Dioscorea deltoidea* Wall.	Dioscoreaceae	2820	Damdama	Kirtha	Tubers	Tubers are crushed to powder form and uses as enhancer of excretion and worm expulsion; Also used in butter as tonic.
31	*Dryopteris juxtapostia* Christ	Pteridaceae	2910	Upper Batakundi	Kwanjay	Young shoots	Young shoots are cooked as pot herb and considered as digestive that help in evacuation of bowel more drastically.
32	*Ephedra gerardiana* Wall. ex Stapf	Ephedraceae	2570	Batakundi R. Station	Ephedra	Whole plant	Powder of the crushed plant and some time its tea is used for TB, asthma, astringent, relaxation of bronchial muscles.
33	*Equisetum arvense* L.	Equisetaceae	2600	Batakundi R. Station	Nari/Bandakey	Aerial parts	Powder prepare from aerial parts are used for bone strengthening, hairs and nail development and weakness caused by TB.
34	*Eremurus himalaicus* Baker	Asphodelaceae	2700	Barrawae	Sheela	Young shoots	Young shoots are cooked and used as digestive.
35	*Euphorbia wallichii* Hook. f.	Euphorbiaceae	3250	Such	Arghamala/Shangla	Latex	Latex is extracted and mixed with milk in small amount and used against worms, to accelerate defecation, promotes circulation and bowel evacuation.
36	*Euphrasia himalayica* Wetts.	Lamiaceae	3170	Lalazar		Whole Plant	Local people cook and use it against cold, cough, sore throat
37	*Fragaria nubicola* Lindl. ex Lacaita	Rosaceae	3200	Jalkhad	Katalmewa	Fruits	Juice of it is considered as anti diarrhoeal, anti dysenteric. Also used in diabetes and sexual diseases.
38	*Fritillaria roylei* Hook. f.	Liliaceae	2950	Dadar Nalah		Bulb	Powder of the dry bulb or in fresh form mixed with butter is used in UTI and to soften and soothe the skin.
39	*Galium aparine* L.	Rubiaceae	3110	Damdama	Goose grass	Whole plant	Its decoction is used in urinary tract infection.
40	*Gentiana kurro* Royle	Gentianaceae	3290	Dabukan	Linkath	Root	Powdered root is used in stomach-ache, as tonic and muscles contraction.
41	*Gentiana moorcroftiana* (Wall. ex G. Don) Airy Shaw	Gentianaceae	3050	Khora	Bhangara	Rhizome	Powder is used to stimulant appetite.
42	*Gentianodes argentia* Omer, Ali & Qaiser	Gentianaceae	3300	Saifalmaluk mountain	Linkathi	Root	Decoction is used in urinary problems.
43	*Geranium nepalense* Sweet.	Geraniaceae	3000	Lalazar	Lijaharri	Whole plant	Rhizome’s powder and decoction of aerial parts are used for the treatment of renal infections and as contraction of uterine muscles.
44	*Geranium wallichianum* D. Don ex. Sweet	Geraniaceae	2920	Barrawae	Lijaahari/Ratan jog/srazela	Rhizome	Boiled powder is used in high blood pressure, uterine diseases and stomach disorders. Also considered as tonic.
45	*Hyoscyamus niger* L.	Solanaceae	2730	Upper Batakundi	Khurasani ajwain	Leaves/seeds	Decoction extracted from boiled leaves in diluted form is used as sedative, and pain killer. Powders of the seeds are used to treat whooping cough.
46	*Hypericum perforatum* L.	Hypericaceae	2540	Naran	Balsana/Shin chae	Whole plnt	Tea prepared of young shoots is used in gastric disorders, respiratory and urinary difficulties. Roots powders are used in irregular menstruation.
47	*Impatiens bicolor* Royle	Balsaminaceae	2700	Lower Batakundi	Gule mehendi/Atraangey	Whole plant	Paste of leaves is used in joint pains. Extract of the plant is regarded as cooling agent and in speeding defecation.
48	*Indigofera heterantha* Wall. ex Brand	Papilionaceae	2680	Saifulmaluk Nalah	Kainthi/Ghvareja	Whole plant	Powder of the root bark and also is used in hepatitis, whooping cough. Its extract is used as dye for blackening of hairs.
49	*Inula grandiflora* Willd.	Asteraceae	2870	Upper Batakundi	Kuth	Rhizome	Both powdered and fresh rhizome is used in gastric disorders, in appetite and as diuretic
50	*Iris hookeriana* Foster	Iridaceae	3340	Besal	Gandechar	Rhizome	Minute amount of powder of dried rhizome is used as speeding defecation and urination and in gall bladder diseases.
51	*Juglans regia* L.	Juglandaceae	2450		Akhor/Ghuz	Fruits, bark	Nuts are believed to use as brain tonic, bark in toothache.
52	*Juniperus communis* L.	Juniperaceae	3550	Such	Gugarr/Bhentri	Berries	Berry powder is considered as enhancer of urination, gas expulsion and stimulant.
53	*Juniperus excelsa* M. Bieb	Juniperaceae	3460	Getidas	Gugarr	Fruits	Fruits are used as urinary, venereal, uterine and digestive troubles as well as gleets.
54	*Leucas cephalotes* (Roth) Spreng.	Papilionaceae	2880	Dabukan	Gomma	Whole plant	Extraction of the plant is used to dispel fever and chills and also used in scabies, cough and cold.
55	*Malva neglecta* Wall.	Malvaceae	2620	Serrian	Sonchal/Panerak	Whole plant	As a local vegetable believed to relinquish bowel and treat dilated veins in swollen anal tissue.
56	*Mentha longifolia* (L.) Hudson.	Lamiaceae	2480	Upper Batakundi	Safid Podina	Whole plant	Fresh leaves and shoots and also its powder are used in sauces with belief of gas expeller and anti diarrhoeal.
57	*Mentha royleana* Benth.	Lamiaceae	2590	Batakundi R. Station	Podina	Leaves	Mixed in green teas and are used in vomiting, as cooling agent and gas expeller.
58	*Nepeta laevigata* (D. Don) Hand.-Mazz.	Lamiaceae	2910	Dadar Nalah	Deijalbhanga/Peesho butay	Whole plant	Powders of the dried plant are used to cure cold, fever and headache.
59	*Onosma bracteatum* Wall.	Boraginaceae	2710	Jakhad	Gowzoban	Whole plant	Powders are taken with water as heart stimulant while decoction is used as anti dandruff.
60	*Origanum vulgare* L.	Lamiaceae	2800	Bans	Jangali majorum	Whole plant	Powder mixed with milk is taken in stomach-ache, antispasmodic. Also taken with milk as antimicrobial and flavouring agent.
61	*Oxyria digyna* (L.) Hill.	Polygonaceae	2940	Khora	Tarwakay	Aerial parts	Young leaves and aerial parts are used as source of vitamin C.
62	*Oxytropis cachemiriana* Camb.	Papilionaceae	3120	Batakundi		Rhizome	Rhizome of the plant is traditionally used as a tooth brush to prevent toothache.
63	*Paeonia emodi* Wall. ex Royle	Paeoniaceae	2730	Naran	Mamekh	Seeds & tubers	Paste prepared from seeds is used in rheumatism. Powdered rhizome is mixed with sweet dishes and used for the treatment of UTI and backache.
64	*Parnassia nubicola* Wall.	Parnassiaceae	3110	Lalazar		Whole plant	Whole plant is cooked as a vegetable (pot herb) and is exercised in digestive disorders.
65	*Pimpinella diversifolia* (Wall.) DC	Apiaceae	2800	Lower Batakundi	Tarpakhi/Watani kaga	Whole plant	Dried plant is crushed to powdered form and used for gas and bowel expulsion. Also used for flavour.
66	*Pinus wallichiana* Jackson	Pinaceae	2720	Lower Batakundi	Sraf	Resin, woods	Resin is considered as diaphoretic, also applied to the cracked (wounded) heels.
67	*Plantago himalaica* Pilger	Plantaginaceae	3230	Barrawae	Jabae	Leaves	Paste prepare from fresh leaves is used in skin problems especially soured feet.
68	*Plantago lanceolata* L.	Plantaginaceae	2950	Upper Batakundi	Ispeghol/Jabae	Leave/ seeds	Decoction of boiled leaves is used in respiratory problems. Seeds are taken with milk to ease digestion.
69	*Plantago major* L.	Plantaginaceae	3000	Dabukan	Ipeghol/Jabae	Root, seeds, leaves	Leaves are cooked and taken orally to cure seasonal fevers. Chopped leaves are used as poultice to cure wounds. Seeds are considered as tonic. Root decoction & infusion is taken as anti dysenteric and leaves decoction in breathing problems.
70	*Podophllum hexandrum* Royle	Podophyllaceae	3080	Khora	Kakorra/Gangorra/	Rhizome & Fruits	A poisonous plant but expert healers use it in a minute amount in mixture with other plants. Its fruit is used to ease bowel movement whilst rhizome is used in the treatment of cancer.
71	*Polygonum aviculare* L.	Polygonaceae	2940	Damdama	Bandakey	Whole plant	Aerial parts of the plant are cooked as pot herb and considered as purgative and emetic
72	*Polygonum plebeium* R. Br	Polygonaceae	3440	Such	Baramol/Noorealam	Root	Root is boiled and mixed with butter locally for stimulate mammary glands; It is also considered to soothes and protects the alimentary canal.
73	*Potentilla anserina* L.	Rosaceae	2820	Lalazar	Spangji	Whole plant	Whole plant is used as anti-diarrhoeal and also in intestinal infections
74	*Primula denticulata* Smith	Primulaceae	3220	Serrian	Mamera	Rhizome	Powdered rhizome mixed with honey is used to cure various eyes disorders.
75	*Prunella vulgaris* L.	Lamiaceae	2910	Upper Batakundi	Ustakhdus	Whole plant	Whole plant both in fresh and dry form is used to relieve respiratory difficulties, in treating joint pains and easing gastric spasm.
76	*Prunus cerasoides* D. Don	Rosaceae	2670	Such	Alubaloo	Bark, fruit	Decoction of the bark is taken in biting and fruit as nerve tonic
77	*Rheum austral* D. Don	Polygonaceae	3450	Saifalmaluk	Chotial	Rhizome & shoots	Leaves and shoots are used as salad for to normalize irregular heart beating, respiratory problems, sore eyes and body strength. Rhizome is cooked and used as wound healing agent and to relive urinary tract disorders.
78	*Rhododendron hypenanthum* Balf.f	Oleaceae	3610	Lalazar peak	Tazak Tusum/Gul namer	Leaves	Fresh leaves of it are used in spices as flavouring agent.
79	*Ribes alpestre* Decne	Grassullariaceae	2720	Batakundi R. Station		Berries	Berry fruits are considered as heart tonic.
80	*Rosa webbiana* Wall. ex Royle	Rosaceae	2900	Besal	Jangali Gulab	Flowerss, bark	Processed flowers (Arq) are used in respiratory problems while bark is used in wounds healing as well as flavour.
81	*Rubus sanctus* Schreber	Rosaceae	3000	Khora	Alish	Whole plant	Fruit is laxative and dysentery; Infusion of leaves and young shoots is used in whooping cough.
82	*Rumex dentatus* L.	Polygonaceae	2540	Lalazar	Shalkhey	Roots & leaves	Root powder is considered to overcome dryness and scaling of the skin.
83	*Rumex nepalensis* Sprenge	Polygonaceae	2670	Lower Batakundi	Ambavati	Roots & leaves	Leaves are used as substitute of Rheum austral whilst its root is believed to ease bowel evacuation.
84	*Salvia lanata* Roxb.	Lamiaceae	2870	Such	Kiyan	Whole plant	Aerial parts are used as vegetable and its root powders are considered to ease bowel evacuation; also used in cough & cold.
85	*Salvia moorcroftiana* Wallich ex Benth	Lamiaceae	2910	Damdama	Kalizarri	Leaves, seeds, roots	Fresh leaves are put in hot ash for a while and then used as poultice for abscesses. Cooked leaves are used in dysentery and colic.
86	*Sambucus weightiana* Wall. ex Wight & Arn	Sambucaceae	2460	Barrawae	Mushkiara	Whole plant	Decoction and powder is used to relieve respiratory difficulties and inflammatory skin conditions.
87	*Saussurea albescens* Hook. f. & Thoms	Asteraceae	3000	Serrian	Kuth	Roots	Roots are cooked in local butters and used as tonic, also use in treatment of stomach as well as pain, and skin diseases.
88	*Silene vulgaris* Garck	Caryophyllaceae	2780	Dabukan	Barra takla	Whole plant	Juice of it is used as digestive, in eye diseases, and is also vaporized to kill or repel pests.
89	*Swertia ciliata* (D. Don ex G. Don) B. L. Burtt	Gentianaceae	2850	Bans	Chirita	Whole plant	Powders are used in irregularity or infrequency of passing faeces as well as stomach burn.
90	*Sysimbrium irio* L.	Brassicaceae	2940	Jalkhad	Khubkalan	Leaves & seeds	Seeds are used in throat & chest infection & ease breathing; Paste of leaves is applied to cure sunburn & enhance skin beauty
91	*Taraxacum officinale* Weber	Asteraceae	2720	Naran	Hand/Gulsag/Booda boodae	Roots	Roots decoction is taken to ease urination and other kidney disorders whilst powders are taken as tonic.
92	*Thymus linearis* Benth.	Lamiaceae	3240	Besal	Bazori/Sperkae/Ban ajwain	Whole plant	Plant is used to make tea, drink, juice to cure stomach & liver complaints; Powder of aerial parts is used in cough.
93	*Trifolium repens* L.	Papilionaceae	2610	Dabukan	Chapatra	Whole plant	Fresh plant is used as worms expulsion (Cattles poison)
94	*Trillidium govanianum* (Wall. ex D. Don) Kunth	Trilliaceae	3370	Saifalmaluk Lake	Tandhi jarri	Roots	Powdered plant is used as body and sexual tonic.
95	*Tussilago farfara* L.	Asteraceae	2990	Batakundi Hills	Funjiwam	Whole plant	Aerial parts are cooked and used in respiratory infections.
96	*Ulmus wallichiana* Planch.	Ulmaceae	2580	Damdama Nalah	Kahey	Bark	Considered highly medicinal for digestive tract diseases.
97	*Valeriana pyrolifolia* Decne	Valerianaceae	3460	Lalazar Peak	Mushkbala/Shangeetae	Rhizome	Powdered rhizome is used to treat spasm and habitual constipation.
98	*Verbascum thapsus* L.	Scrophulariaceae	2980	Naran	Kharghvag/Jangali tamakoo	Whole plant	Root’s powder is considered as aphrodisiac; leaves, paste is used in skin problems; leaves are also smoked to induce sedation by reducing irritability or excitement.
99	*Viburnum cotinifolium* D. Don	Caprifoliaceae	2630	Naran	Taliana	Fruits	Fruits are taken for reducing uterine irritability and stopping bleeding usually by female
100	*Viburnum grandiflorum* Wall. ex DC.	Caprifoliaceae	2680	Naran	Guch	Fruits	Fruits are used to ease gastric spasms and uterine irritability.
101	*Viola canescens* Wall. ex Roxb.	Violaceae	3020	Jalkhad	Gule banafsha	Whole plant	Young shoots are used to promote circulation, dispels fever and chills, relieves muscle tension whilst decoction & infusion is used in sore throat.

### Plants’ parts used and their preparation

**Figure 3:**
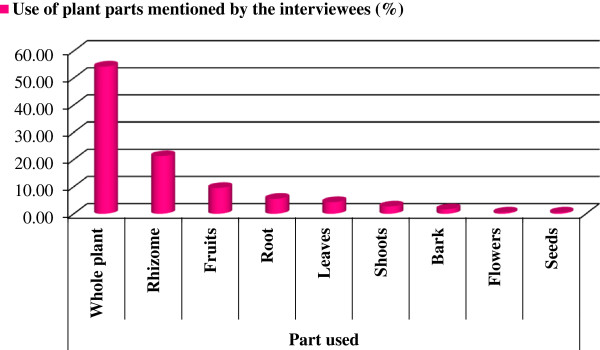
Parts of plant used for medicinal purposes.

**Figure 4:**
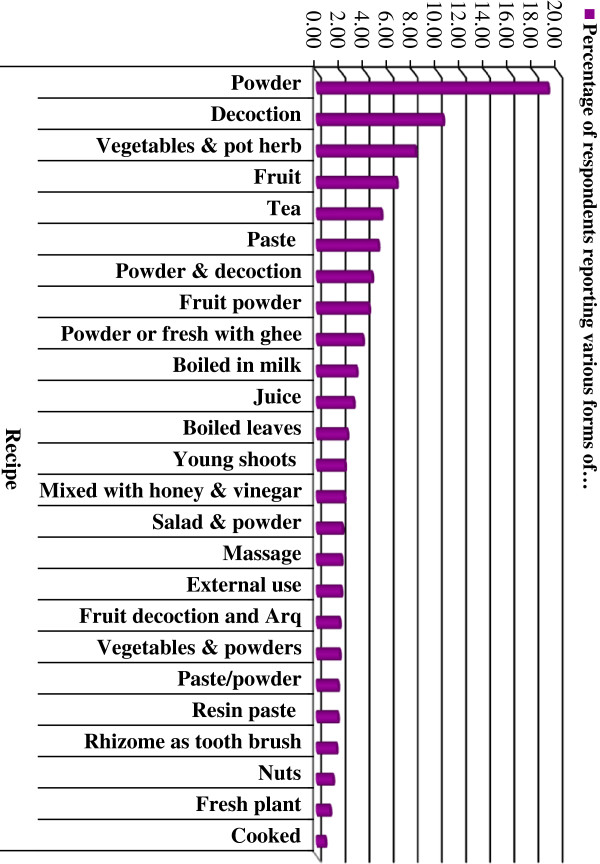
Recipes for medicinal plants reported by interviewees (%).

The interview results indicate that whole plants are used in 54% of treatments followed by rhizomes (21%), fruits (9.5%) and roots (5.5%). Bark, flowers and seeds were used less frequently. Most of the plants used are hemi-cryptophytes and geophytes and fewer are woody (phanerophytes and chamaephytes) or therophytes (Figure 3). Whole plants or plant parts are utilized in various forms in traditional herbal recipes. In the majority of recipes, they are in the form of powder (19%) followed by decoction + infusion (10.5%) (Figure 4).

### Therapeutic uses

**Figure 5:**
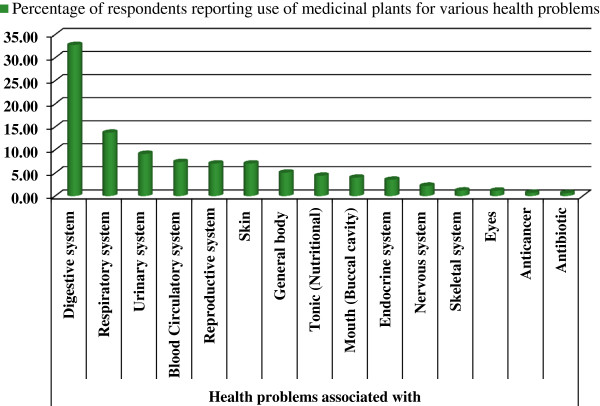
Medicinal use categories mentioned by respondents for the treatment of different categories of disease or other ailments.

**Figure 6:**
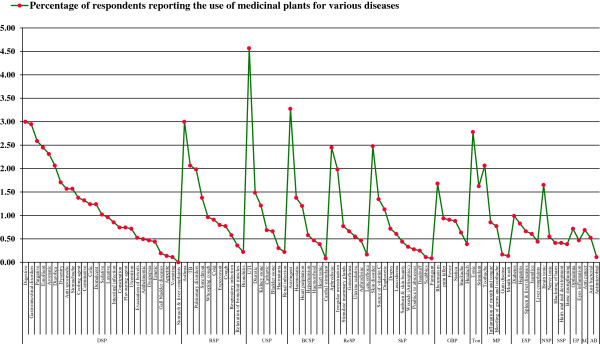
**Medicinal plant uses for treating different diseases & ailments.** DSP = Digestive System’s Problems; RSP = Respiratory System’s Problems; USP = Urinary System’s Problems; BCSP = Blood Circulatory System’s Problems; ReSP = Reproductive System’s Problems; SkP = Skin Problems; GBP = General Body Problems; Ton = Tonic; MP = Mouth Problems; ESP = Endocrine System Problems; NSP = Nervous System’s Problem; SSP = Skeleton System’s problems; EP = Eyes problems; AC = Anti Cancer AB = Antibacterial.

The results of the questionnaires analysis reveal 97 prominent remedial uses of medicinal plants, which were divided into 15 major categories based on the ailment of a specific human system, being treated with. The largest number of ailments cured with medicinal plants are associated with the digestive system (32.76% responses) followed by those associated with the respiratory and urinary systems (13.72% and 9.13% respectively). The percentage of ailments associated with the blood circulatory and reproductive systems and the skin were 7.37%, 7.04% and 7.03%, respectively. In terms of a single problem of a specific system, the urinary tract infection (UTI) was mentioned on top; treated with medicinal plants followed by asthma and gastric problems. The other diseases related with general body, endocrine system, nervous system, mouth and eyes etc. were considered each by 5% or less than 5% respondents. Figure 5 and 6 visualise the results of the specific diseases of the human system cured with medicinal plants as mentioned by interviewees, whilst a detailed summary of the species along with a list of the specific diseases is presented in Table 2.

## Discussion

### Role of native plants in supporting human livelihoods and well being

Findings of this paper signify the relationship between the provisioning ecosystem services of vegetation and human well-being in the study area. The questionnaire analyses indicate that the people of the Naran Valley possess valuable knowledge of natural plant biodiversity and the services it can provide are immensely important to them. There was a variation in knowledge at individual level depending upon the relation between the person and the specific plants species or group which he/she prioritizes for certain uses which is reported in number of other studies also [29,30]. Nevertheless, this study has been able to demonstrate that plants are used to support a wide range of livelihood activities in the study area, and particularly as a source of traditional medicines. Furthermore, plant biodiversity of the region provide timber, fuel, medicines, food, fodder, grazing and others services to the indigenous communities. However extensive uses of natural vegetation in the past have decreased the provisioning services. Local residents especially the older generation prefer to live in the valley because of the existing provisioning ecosystem services and their traditional ethno-ecological knowledge. However, the new generation tend to leave those rural spaces in search of education, facilities and easy modern life [31].

### Medicinal plant resources

The use of plants to cure diseases is as old as human history. Around 20% of the plant species of the world are estimated to be used in health care systems [32]. Medicinal plants play an important role in the traditional health care systems of this region also. A few of the species found in the region, i.e. *Dioscorea deltoidea, Podophyllum hexandrum, Berberis pseudoumbellata, Cypripedium cordigerum* and *Dactylorhiza hatagirea,* are listed on the CITES appendix II (the Convention on International Trade in Endangered Species of Wild Fauna and Flora). One or more of these species were also reported in few other studies from other Himalayan and the Hindu Kush areas [17,33]. Some other medicinal species are endemic to the Himalayas and hence should also be given attention in order to ensure sustainable use e.g., *Cedrus deodara* and *Aesculus indica,* whilst some are rare in the region e.g., *Aconitum heterophyllum, Aconitum violaceum, Ephedra gerardiana, Hypericum perforatum, Indigofera heterantha, Paeonia emodi,* and *Ulmus wallichiana*. In addition to medicinal uses, these species provide other services like timber fuel and grazing etc. Numbers of the remote valleys in the Himalayas have not been studied specifically for the ecosystem services and plant medicinal uses though indigenous inhabitants of these areas have a long established system of health care and cure with available medicinal plant resources. The elder people have more accurate knowledge about the parts and recipes than the young which improve the effectiveness of medicinal plants. Similar trend was also reported by other ethnobotanists in the Southern Ethiopia and Hindu Kush region [34,35]. It is important to recognise that unsustainable collection of medicinal plants is one of the main causes of plant population decline. Increasing human population, extensive grazing, habitats losses, multipurpose collection and carelessness are the other factors. All such ecological as well as cultural matters need to be documented and addressed while designing management, preservation and conservation strategies. Results of this paper also demonstrate that most of the plants are either used as a whole or its parts like roots and rhizomes distinctively which are also alarming signals against the sustainable use of this highly valued plant biodiversity. In addition marketing of certain species indicate probable threats information about their conservation which also suggests that number of these economically important species can be domesticated and propagated in protected places for marketing purposes. Interestingly, most of the less abundant species and families are utilized frequently by the locals. For example, 31 small families with only 1 or 2 species are all used medicinally in the area. These findings not only prove that peoples utilize the plants according to their traditional knowledge and not their abundance but also indicate the rarity of such taxa in the near future.

### Indigenous knowledge as a cultural asset

Rapid technological and economic development has brought ecological and social changes all over the world. Cultural changes even take place in remote rural societies due to their increasing interactions with modern urban cities. Subsequently, knowledge about the use of plant resources, as well as the plant wealth itself, is declining in a number of regions [36,37]. The present study also reveals a decrease in indigenous knowledge and changes in attitudes regarding health-giving flora among the younger generation. This phenomenon is confirmed from the study of [38] on the Pakistani migrants in Bradford UK. This and other similar studies, further communicate the extinction of traditional knowledge in modern societies. Indigenous people, although the possessor of traditional knowledge have no proper training in sustainable ways of plant collection, post collection care and processing and usually waste a considerable amount of medicinal plants. Such sort of unwise practices over a long time can cause a reduction in plant biodiversity in general and of plant species providing provisioning services in particular [39,40]. It is therefore, suggested to recruit ethno-ecologists and experts to train the local people for the sustainable utilization of medicinal plant resources. Some of the problems associated with medicinal plant resources can be overcome through research on domestic growth of medicinal plants and development of processing techniques among the people. In this recent millennium, present and number of other research studies suggest urgent call for the preservation of both long-established remedial knowledge and medicinal plant resources in the developing world, particularly in the Himalayas [17,41-44]. Furthermore, long-established knowledge about the medicinal values of plants has contributed a lot in the past in production and synthesis of synthetic drugs and market values. It has played and still plays a remarkable role in solving health related problems especially in undeveloped and remote parts of the world. A number of issues were identified during the present project. These include documentation of the traditional knowledge; intellectual property rights of the locals, trainings about the sustainable use of the available resources and use of the traditional knowledge for conservation which can be addressed in the future.

## Competing interests

SMK involved in formulating study design, questionnaire, field work, data collection and compilation of 1st draft of this paper. SP, HA and DMH planned questionnaire and supervised the project. HS gathered relevant literature. ZU identified most of the plant species in the field. MA processed and preserved the herbarium specimens. All the authors have read and approved the final submission of the paper.

## Authors’ contributions

The authors articulate that they have no competing interest.
